# Stochastic Nature of Fascia: From Layered Pedagogical Artifact to Morphogenetic Reality in Clinical Anatomy

**DOI:** 10.3390/life15121924

**Published:** 2025-12-16

**Authors:** John Sharkey, Karen B. Kirkness

**Affiliations:** 1 Irish College of Osteopathic Medicine, National Training Centre, DO7 F6CR Dublin, Ireland; 2 Health Professions Education Unit, Hull York Medical School, York YO10 5DD, UK

**Keywords:** connective tissue, fascia, clinical anatomy, medical education, endoscopy, hyaluronan, calcium, mechanobiology, morphogenetic fields, stochastic, deterministic

## Abstract

Fascia research suffers from definitional fragmentation, with no universal agreement about what fascia actually is, why it matters, or how to define it. Researchers often pursue lines of inquiry based on their existing expertise, yet traditional and newer approaches that might resolve these issues frequently conflict. To address this challenge, the authors use a hermeneutic framework to integrate their combined half century of anatomical experience with a narrative literature synthesis. They propose that fascia functions as a stochastic morphogenetic field rather than a discrete anatomical system, a stochastic process displaying opportunistic dynamics at atomic, molecular, and cellular scales that produces deterministic mechanical properties at macroscopic tissue levels. Four key conclusions emerge: (1) anatomical “virtual spaces” are hyaluronic acid (HA)–tissue manifolds tightly coupled with calcium coordination; (2) fascia functions as a stochastic morphogenetic field where clinically and educationally relevant deterministic patterns emerge; (3) a conceptual framework for context-flexible fascial nomenclature; (4) hermeneutic approaches enable synthesis across theoretical domains. The conclusions support the understanding of HA-mediated EMT/MET plasticity and its “Go or Grow” phenotypes as central conduits for both healing and cancer progression. Understanding the stochastic nature of fascia is thus essential for physicians as well as clinicians in the allied health setting. Optimal fascia-aware movement and manual therapy interventions are those that recognize fascia as a self-adapting morphogenetic field.

## 1. Introduction

### 1.1. Fascia: The Tissue of Water

Fascia forms a fluidic, body-wide continuum that surrounds, interpenetrates, and connects all bodily tissues [1]. This tissue provides both support and an integrated substrate within which body systems develop and function together [2,3,4]. This network is not merely structural; it plays active roles in movement and force transmission [5,6]. In this narrative review, we focus on fascia as a mediator of body-wide biomolecular integration through its responsive intra- and extracellular matrix (ECM).

The continuity of fascia in its fibrous and gel-like expressions is now well established; however, the probabilistic links between its molecular and macro-level behavior remain underexplored in the literature. We argue that fascia resists definition because of this disconnect between micro and macro. We propose that it functions as a reciprocal morphogenetic field, which explains emergent clinical patterns.

This relationship between micro and macro is measurable in the relative concentration of hyaluronan (HA) found across various body regions. HA is a high-molecular-weight, linear glycosaminoglycan. This complex sugar molecule is composed of repeating, two-sugar building blocks forming an unbranched polysaccharide chain. Its highly hydrophilic nature allows HA to bind up to 1000 times its weight in water [7,8,9].

The gel-like qualities arising from HA’s water binding contribute to the characteristic tissue hydration and viscoelasticity observed in the ECM [10,11]. HA is thus commonly said to “lubricate” tissues, but biophysically, this effect arises from its hydration-dependent viscoelasticity rather than simple mechanical lubrication. We argue that distinctions such as this, rather than merely pedantic, are crucial for advancing our understanding of fascia.

HA regulation in fascial planes is a stochastic mesenchymal cell activity. Individual cells respond to apparent randomness (stochasticity), but tissue-wide patterns are predictable (deterministic)—like how random raindrops create predictable puddles. In the body, specialized cells like fasciacytes stochastically produce and remodel HA within the ECM to maintain tissue gliding and structural integrity. We identify the calcium-HAS2 axis (CHA) as a molecular mechanism through which this stochastic-deterministic organization emerges.

**Figure 1:**
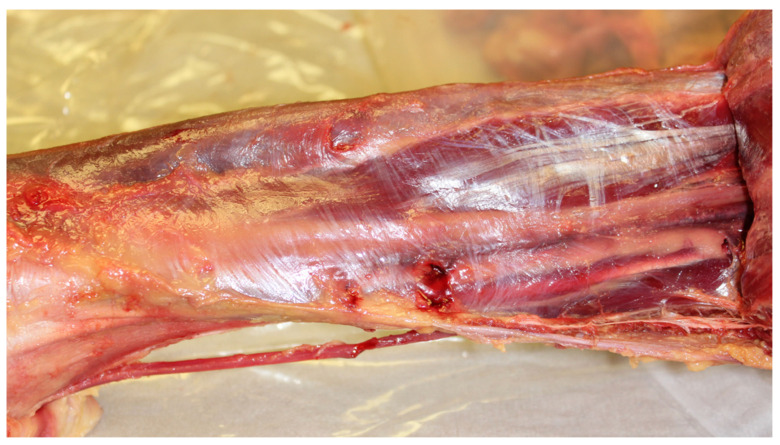
Gastrocnemius and soleus muscles reflected to expose the deep fascia and underlying structures. The deep fascia visible here exemplifies a fascial plane manifold with elevated HA concentration, reflecting increased biochemical activity and enhanced gliding mobility. This functional specialization corresponds to the biomechanical demands of walking and lower limb movement. Image: Sharkey, J. 2023.

The deep fascia of the lower leg (Figure 1), for example, is known for elevated HA levels compared to less mobile fascia. High HA levels support enhanced gliding and biomechanical function, although exact quantitative comparisons to other regions are limited [12]. Peripheral nerve injury studies in rats show that HA concentration in lower limb muscles and thoracolumbar fascia decreases significantly after injury, indicating the importance of HA in maintaining normal tissue function [12]. The lower leg fascia and muscles exhibit relatively high HA concentrations compared to some other muscle fasciae, consistent with their role in facilitating mobility and load-bearing during locomotion.

### 1.2. Developmental Origins of Fascia

Understanding the HA-regulating behavior of fascia requires an appreciation of its developmental origins. Fascia develops from cells derived primarily from mesodermal mesenchyme. Mesenchymal cells differentiate into various supportive cell types including fibroblasts, fasciacytes, osteoblasts, chondrocytes, and endothelial progenitor cells, among others, all of which secrete HA proportionately. Fibroblasts are well-known for secreting HA and collagen, contributing to the ECM, while osteoblasts primarily secrete bone matrix components but can also be influenced by HA during differentiation [13].

Mesenchymal stem cells (MSCs) themselves have a strong intrinsic capacity to synthesize and secrete HA, often releasing HA-coated extracellular vesicles that participate in tissue regeneration and remodeling [14]. HA plays a significant role in promoting differentiation of MSCs into specialized cells such as alveolar type II pneumocytes and chondrocytes, indicating that HA secretion is linked to their differentiation processes [15,16,17]. However, not all mesenchymal-derived cells secrete HA to the same degree. For example, osteoblasts secrete less HA compared to fibroblasts or MSCs, where HA secretion is more prominent and functionally important [13,14].

Mesenchyme is volumetrically three-dimensional and functionally multidimensional, providing the cellular substrate from which the body’s entire fascial network emerges. During development, mesenchymal cells populate distinct anatomical regions and differentiate into specialized fascial tissues at interfaces referred to as ‘fascial planes.’ This article proposes that these context-dependent pathways are shaped by local mechanical forces, biochemical gradients, and tissue-specific signaling environments.

### 1.3. Stochastic-Deterministic Behavior in Fascia

Fibroblasts exhibit stochastic behavior at the cellular level in several ways, including variability in their proliferative potential. Daughter cells from a single mitosis differ significantly in proliferation capacity, suggesting a stochastic mechanism underlying cellular aging [18]. The stochastic nature of gene activation also underlies variability in fibroblast responses during processes like activation, proliferation, and differentiation, which are critical in tissue remodeling and fibrosis [19,20]. Fibroblast gene activation is a probabilistic process influenced by multiple regulatory steps and signaling interactions, leading to diverse cellular outcomes even within genetically identical populations [19,21,22]. This stochastic gene activation contributes to heterogeneity in fibroblast function and behavior, feeding back to the links with HA regulation explored in this narrative review.

Fascia’s dual nature reflects how seemingly random cellular activities collectively produce predictable mechanical and structural responses in fascia tissue, as shown in studies modeling fascia’s mechanical properties under various tension states [23]. The term “stochastic” derives from the Greek *stokhastikos*, meaning “to aim” or “to guess”—reflecting processes governed by probability rather than certainty. Processes or models that are fundamentally probabilistic are those governed by the laws of probability, rather than being purely random in the sense of complete unpredictability or lack of structure. This approach allows the model to predict a range of possible outcomes, each with an associated likelihood, rather than a single deterministic result [24,25].

Stochastic processes are those that are inherently random but can be described statistically; outcomes are governed by probability distributions. Cellular events are not strictly random, but fundamentally opportunistic at the molecular level—behaving responsively to changing conditions, not strictly governed by chance or fixed rules. While these events are subject to constraints and modulation by feedback loops, they retain an element of unpredictability. The variability is not unbounded or chaotic; it is constrained by the underlying probability distributions and system parameters, ensuring that predictions remain within plausible, biologically or physically meaningful limits [26]. Our central argument is that stochasticity in fascia (arising from opportunistic variation at the nano scale) results in constrained, probabilistic, but not strictly deterministic, behavior.

### 1.4. Aims and Scope

This perspective article addresses a fundamental question: How can fascia be reframed as a stochastic morphogenetic field? While numerous attempts have been made to define fascia, few have explicitly recognized why this tissue resists singular definition. We propose that fascia’s inherent stochastic-deterministic duality fundamentally resists conventional single-definition approaches.

This work makes three core contributions. First, we reframe fascial “virtual spaces” as HA manifolds—dynamic tissue interfaces rather than empty cavities. It bears mentioning that this review focuses on manifolds that maintain surface orientability. Second, we highlight the recently proposed Ca^2+^-HAS2 axis (CHA) as a mechanobiological framework linking stochasticity to tissue-level organization. Third, we present a context-flexible nomenclature tool that adapts terminology for educational, clinical, and research contexts.

Our intended audiences span three communities: researchers investigating fascial biomechanics and morphogenesis, clinicians requiring anatomically precise yet functionally relevant terminology, and educators seeking frameworks that bridge traditional anatomy with contemporary tissue science. By integrating evidence from embryology, mechanobiology, computational anatomy, and clinical practice, we demonstrate how opportunistic cellular events at the microscale produce predictable tissue patterns at the macroscale.

### 1.5. The Paradigm Shift

Researchers, including the authors of this article, have called for paradigm shifts in how we understand fascia for decades [27,28,29,30,31,32,33,34]. Yet no comprehensive perspective has unified the mathematical manifold framework with stochastic organization theory and morphogenetic field concepts. This article uniquely integrates clinical anatomy with stochastic HA dynamics into a cohesive framework challenging traditional nomenclature while maintaining clinical relevance.

## 2. Methods

### 2.1. Hermeneutic Approach

STEM fields traditionally emphasize objective measurement and quantifiable data. However, emerging evidence supports hermeneutic (interpretive) philosophy for deepening understanding of complex phenomena resisting purely reductionist approaches [35,36]. This philosophy challenges the strict subject-object divide by recognizing scientists and learners as interpreters engaged in a continuous hermeneutic circle, where pre-understandings and new insights fuse to expand horizons of knowledge [37,38]. In natural sciences such as anatomy, hermeneutics incorporates historicality, culture, and tradition into scientific realism. The authors’ stance is that scientific knowledge is not only about objective measurement but also about meaning-making within a lifeworld context [35,36].

### 2.2. Literature Exploration Scope

Following hermeneutic meta-synthesis principles, this perspective engaged in dialogical process with texts, applying Gadamer’s concepts of the hermeneutic circle and fusion of horizons [37]. Over a one-year period, the authors conducted purposive explorations of peer-reviewed databases (PubMed, ScienceDirect) focusing on anatomical “virtual spaces” as continuous connective tissue manifolds. Search terms included “stochasticity,” “fascia,” “deterministic,” “anatomical manifolds,” “mesenchymal cell signaling,” “fascial planes,” “gliding versus sliding” and “anatomy virtual spaces.”

AI search tools were used iteratively in conjunction with manual database searches. A comprehensive literature search was conducted across over 170 million research papers using Consensus, a tool that aggregates content from Semantic Scholar, PubMed, and other databases. The search strategy focused on foundational concepts, clinical outcomes, anatomical and biomechanical properties, and evolving clinical practices related to fascial continuity. The search identified 1020 papers (*n* = 1019 from 20 Consensus searches; *n* = 1 from citation graph exploration). After removing papers with missing abstracts and duplicates, 546 papers were screened. Papers with low semantic relevance were excluded, yielding 362 eligible papers. Following final quality ranking, the top 23 highest-quality papers were included in a review that was drawn into the iterative process.

In parallel, the authors drew upon their collective experience with fresh and Thiel soft-fixed human cadaver dissections. Both independently and in collaboration, the authors have developed familiarity with fascial planes and anatomical virtual spaces. Their literature search and analysis were informed by their pre-existing phenomenological understanding of such planes and spaces. All threads of research were woven into one living document refined over several rounds of analysis. Literature exploration was guided by classical anatomical models and emerging continuity theories, tracing connections between anatomical structure, clinical relevance, and mathematical abstraction. This collaborative approach examined virtual anatomical spaces and their relation to pathology, fluid dynamics, and surgical outcomes, enabling novel insights to emerge through dialog.

All anatomical images included in this manuscript are original material obtained by the authors during institutional cadaveric dissection sessions conducted for educational purposes. In accordance with Italian legislation, the observational use of cadaveric material for teaching and research does not require additional ethical committee approval when no experimental procedures are performed. No identifiable donor information is present, and all legal and ethical requirements for the respectful use of human donors were fully observed.

## 3. Results: Narrative Synthesis

### 3.1. Go or Grow: EMT-MET as a Lifelong Dynamical Feedback Loop

The results of our review start at the beginning: with embryology. The epithelial–mesenchymal transition (EMT) and its reverse process, mesenchymal-to-epithelial transition (MET), broadly characterize development. These bidirectional cellular transformations continue throughout life. EMT/MET is shorthand for the fundamental cellular plasticity known as “Go or Grow”: cells can either proliferate (“Grow”—building tissue, dividing, remaining stationary) or migrate (“Go”—moving, invading, remodeling the ECM [39].

In EMT, epithelial cells lose epithelial characteristics, such as apical-basal polarity and tight cell–cell adhesion. They acquire mesenchymal “Go” traits including motility, invasiveness, and matrix remodeling capacity [40,41,42,43]. Conversely, in MET, mesenchymal cells (including fibroblasts) acquire epithelial “Grow” characteristics: organized polarity, structured adhesion complexes, reduced motility, and increased proliferative capacity [44,45,46].

Critically, these transitions are not binary switches but exist on a stochastic spectrum with multiple intermediate and hybrid states. Cells can simultaneously exhibit both “Go” and “Grow” characteristics, creating a continuum of cellular phenotypes [43,47,48]. This plasticity governs not only mesenchymal cells during development and repair but also cancer cells during metastasis, stem cells during mobilization, and other regenerative processes. In fascial tissues, this EMT/MET plasticity allows cells to dynamically adapt their behavior (Table 1). They switch between migratory matrix-remodeling states and stationary matrix-producing states in response to mechanical forces, injury, and changing tissue demands.

### 3.2. Fasciacytes: A Distinct Mesenchymal Cell Type with HA Specialization

A key concept is that mesenchymal cells possess diverse differentiation potentials that depend on context and local signals [53,54]. Where they “go” has everything to do with what kind of cell they “grow” into, developing specialized behavior to suit their role. Recent research establishes fasciacytes as a distinct mesenchymal cell type specialized for fascia, with unique molecular and functional properties that set them apart from traditional fibroblasts [55,56,57].

The defining feature of fasciacytes is exceptional HA production and strong expression of HAS2, the key HA-synthesizing enzyme—far exceeding fibroblast HA output [55,56]. Single-cell RNA sequencing has revealed significant heterogeneity among fascia-associated cells, identifying unique clusters with distinct gene expression profiles and secretory functions, including fasciacyte populations specialized for ECM production and HA secretion [56,57,58]. This molecular evidence positions fasciacytes not merely as specialized fibroblasts but as a functionally distinct cell lineage optimized for the biochemical demands of fascial tissue. As a relatively recent discovery, fasciacytes have not yet amassed the same level of experimental evidence as other mesenchymal cells.

Across cell types, HA synthesis and remodeling are closely linked to EMT processes, as HA and HAS2 are upregulated during EMT, promoting cell migration and plasticity [59]. EMT-inducing transcription factors like ZEB1 regulate HA network components, including HAS2 and HA-binding proteins, facilitating ECM remodeling that supports mesenchymal traits and motility [51,59]. This suggests that fasciacytes, like other mesenchymal cells, exhibit high EMT/MET plasticity. This agility contributes to their dynamic roles in tissue maintenance, repair, fibrosis, and pathological conditions [51,56,57].

### 3.3. HA Distribution Across Scales

In fascial tissues, this plasticity gives rise to changeable identities—oscillating between migratory matrix-remodeling states (“Go”) and stationary HA-producing states (“Grow”) in response to mechanical forces, biochemical signals, injury, and repair demands [60]. These cells operate in a stochastic environment, optimized for shifting their molecular profile and functional behavior as tissue conditions change, with HA regulation serving as both a marker and modulator of their phenotypic state. Yet this cellular-level plasticity represents just one scale in a much larger organizational hierarchy—a multi-scale waterway system in which HA flows, accumulates, and communicates across the entire body.

To understand this system, we must ask: where in the body are concentrations of HA at their highest, and what does this distribution reveal about scale-dependent organization? At the organ scale, skin contains about half of the body’s total HA, distributed between the dermis and epidermis, essential for hydration and elasticity [10,61,62,63]. At the joint scale, synovial fluid has the highest HA concentration by volume, crucial for viscoelasticity and shock absorption [64,65]. Between these scales lie the fascial planes—an intermediate level where HA concentrations respond dynamically to the mechanical demands of tissue gliding and force transmission [66,67].

### 3.4. ”Waterways”: Fascial Planes as Communicative Channels

This pattern reveals a fundamental principle: HA concentration scales with movement demands at every level of organization. From molecular hydration shells around individual HA chains, to cellular microenvironments where fasciacytes modulate their secretory activity, to tissue-level fascial planes that facilitate gliding, to organ systems like joints and skin that bear the body’s mechanical loads: each scale exhibits HA regulation tuned to its specific functional requirements. The presence and regulation of HA in fascial planes are critical for normal movement and may have evolved to meet the mechanical demands of different body regions [67]. Skin, joints, eyes, and fascial planes all carry different movement requirements, yet each secretes HA at the level required, all feeding into an interconnected gel-like substrate.

A useful analogy: as all waterways eventually flow to the sea, this bodywide continuum of HA creates an inherently communicative network. In the body, water does not flow *freely*; it is molecularly bound to the HA strands. Intra-tissue endoscopy has revealed a ubiquitous, adaptable fibrillar network (likely corresponding to fascial tissue) of fluid-filled microvolumes. What Guimberteau has termed “microvacuoles” [28] and Theise refers to as the “interstitium” [68] are amongst the finest tributaries of this system: molecular-scale channels where hydrated HA “flows.” This network extends from the skin surface to the cellular level, enabling tissue continuity and gliding with minimal distortion [69]. These microscale waterways converge into fascial planes, which serve as the major rivers of the system, channeling mechanical signals and biochemical information across tissue boundaries.

Our interpretation is that fascial planes do not just allow for gliding; they exist because of gliding, and the gliding feeds back to reinforce their function as conduits in this multi-scale network. Understanding fascial planes as one critical level in this hierarchical waterway system—positioned between cellular microenvironments and organ-scale structures—sets the stage for a deeper visualization of their geometry and topology. In the next section, we explore how fascial planes can be conceptualized as manifolds.

### 3.5. Fascia Planes and the Manifold Model

**Figure 2:**
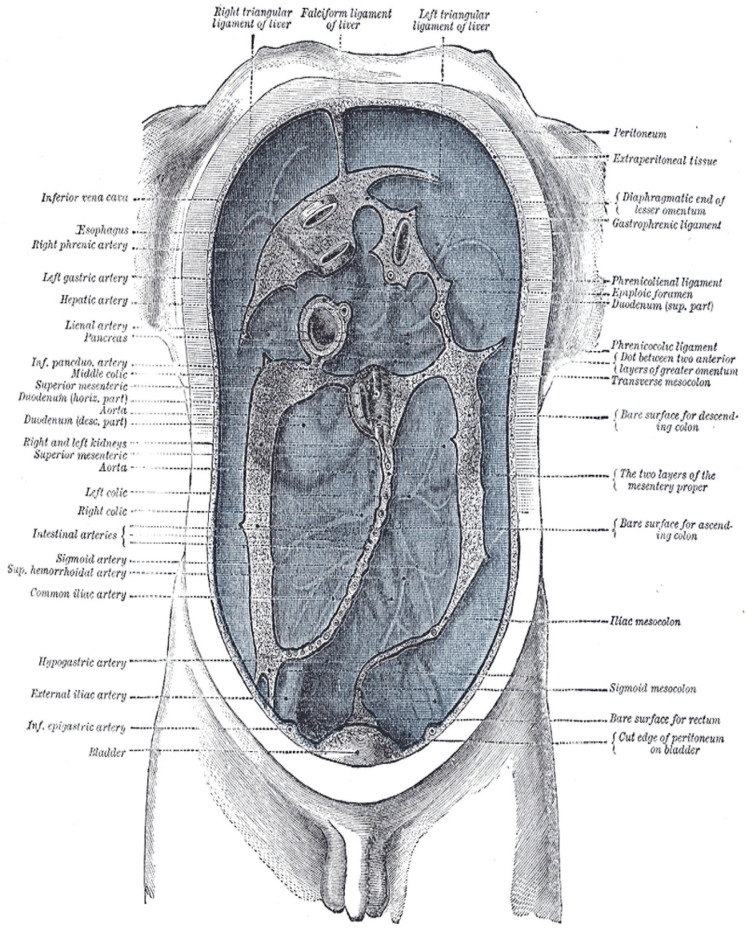
Delépine’s diagram illustrating the lines along which the peritoneum leaves the abdominal wall to invest the viscera. This classic anatomical diagram reveals the peritoneum as a continuous surface with complex folding patterns—exemplifying manifold topology where a single sheet creates multiple compartments, mesenteries, and reflections through embryological development. The diagram visualizes how local flat regions transition through folds into globally curved, interconnected spaces—the defining characteristic of an anatomical manifold. From Henry Gray (1918) https://www.biodiversitylibrary.org/item/60234 (acccessed on 1 October 2025) Anatomy of the Human Body, Plate 1040. Illustration by Henry Vandyke Carter (1858). Public domain.

Just as Earth’s surface appears flat locally but curves globally, fascial planes appear as discrete layers locally but form continuous surfaces—this is manifold geometry. The peritoneum provides a clear anatomical example of a manifold (Figure 2). This 2-dimensional continuous surface locally appears flat but globally forms a complex, curved topology. It is the largest serous membrane in the body, often described as a single continuous sheet that lines the abdominal cavity (parietal) and covers organs (visceral). Through embryological folding, it emerges as a continuum of reflections, ligaments, mesenteries, and compartments [70,71,72]. At any small region, the peritoneum appears Euclidean (flat) but globally exhibits complex curvature with multiple interconnected subspaces—the mathematical definition of a manifold [73,74]. This manifold structure underlies both normal physiological function and pathological spread patterns, illustrating how fascial and serous tissue organization transcends simple layered models.

We argue that fascial planes function as a dynamic, self-organizing manifold at the tissue level, providing a framework to understand the biochemical feedback loops that regulate tissue behavior. This network acts as both a scaffolding for cells and a force absorption system, explaining how virtual “spaces” in anatomy correspond to HA-tissue interfaces, which unify the concepts of anatomical continuity and differentiation. Fascia’s self-regulatory nature aligns with organizing principles from biology and physics, supporting its role in tissue form and function across the body [75].

Surgical anatomy studies further demonstrate that fascial planes define compartments with distinct functions, reinforcing the idea of fascia as a continuous yet compartmentalized manifold that guides both structure and movement [76]. The manifold concept of fascia integrates structural, biochemical, and mechanical perspectives. As a comprehensive model for living tissue organization and behavior, the manifold topology brings the HA substrate into a continuous feedback loop that we may encounter in dissection. Paradoxically, the standard tool for observing this continuity is, of course, the scalpel.

### 3.6. Superficial Fascia

**Figure 3:**
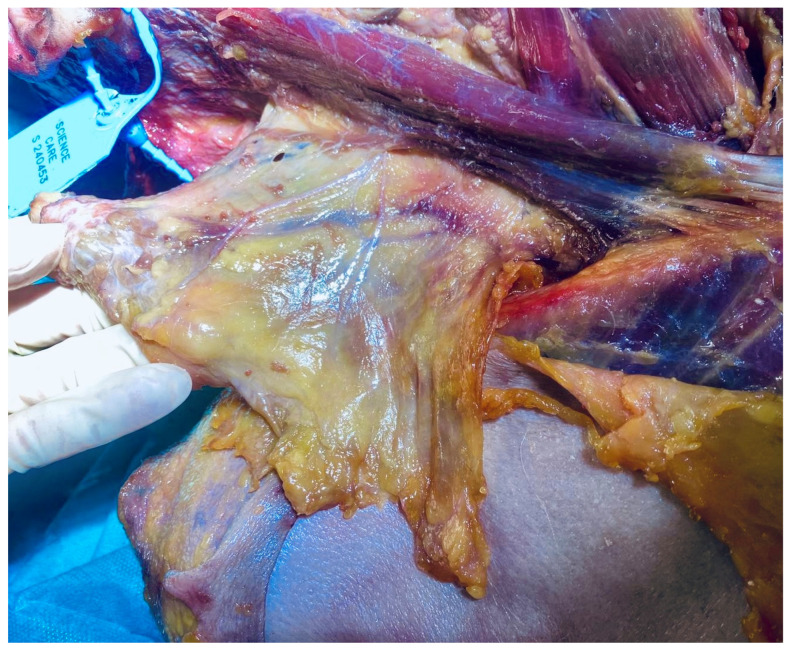
Fascia-focused dissection of the superficial cervical fascia including the platysma (fresh-frozen cadaveric specimen). Image: Sharkey, J. 2024.

We now turn to evidence from such encounters as informed by the literature. The superficial cervical fascia (Figure 3) is a distinct fascial plane located within the subcutaneous tissue of the neck. It contains a rich intrinsic neural network that may contribute to chronic cervical myofascial pain and related symptoms such as cervicogenic dizziness and paresthesias [77]. Recent research has revealed the superficial fascia as a crucial sensory interface, far beyond its traditional classification as mere subcutaneous tissue (Fede et al., 2025 [78]; Pirri et al., 2023 [79]). Characterized by high HA concentration and dense mechanoreceptor networks, it serves as a critical proprioceptive organ, mediating body awareness and load transmission between skin and deeper musculoskeletal structures (Pirri et al., 2023 [79]). This rich mechanosensory environment makes the superficial fascia an ideal site for exploring how EMT/MET directly influences biochemical signaling and tissue homeostasis.

The superficial fascia is composed of cellular and ECM components, including collagen, elastin, and HA, which contribute to its thickness, elasticity, and capacity to facilitate gliding. It plays a key role in the transmission of sensory signals and potentially serves as a target for ultrasound-guided interventions like hydro-dissection to relieve myofascial pain resistant to conservative treatments. Blocks targeting the superficial cervical plexus and adjacent fascial planes provide effective analgesia for surgeries involving the clavicle and neck. Advantages of blocks here include preserving motor function and reducing complications such as diaphragmatic paralysis [80,81]. Recent reviews emphasize the need to better understand the superficial fascia to optimize both pain management and surgical approaches in the cervical region [78,82].

### 3.7. Anatomical ‘Virtual Spaces’ as HA-Rich Tissue Interfaces

This section argues that anatomical “spaces” are never truly empty—they are HA-modulated tissue interfaces [83]. Traditional nomenclature misleadingly implies that regions such as the retroperitoneal space, potential peritoneal cavities, and interfascial planes represent voids. Evidence demonstrates these regions are specialized zones of fascial architecture where connective tissue organization adapts to functional demands. This view aligns with emerging perspectives in surgical literature recognizing that anatomical “spaces” are continuous with surrounding fascial networks rather than discrete voids [84,85,86].

#### 3.7.1. The Pouch of Douglas and Pelvic Fascial Architecture

The rectouterine pouch (Pouch of Douglas) exemplifies this principle. It is a continuous HA and glycosaminoglycan (GAG)-rich areolar connective tissue interface that forms an uninterrupted continuum between the rectum and posterior uterine wall [87]. This fascial interface represents a dynamic ECM environment where HA and GAGs create a cohesive tissue plane that extends throughout the pelvic cavity [88,89]. The continuous nature of this GAG-rich interface is fundamental to understanding both normal pelvic physiology and the pathological processes that traverse these anatomical boundaries [88,90].

This HA-GAG continuum serves multiple critical physiological functions within the pelvic cavity. The high water-binding capacity of HA cushions and provides gliding adjacency between organs, facilitating smooth visceral movement during normal physiological activities such as bowel peristalsis, bladder filling, and uterine changes throughout the menstrual cycle [91]. Beyond mechanical support, this viscoelastic matrix actively supports cell migration during tissue repair processes and modulates inflammatory responses through complex signaling pathways [92]. The structural integrity of pelvic organs depends on this continuous fascial interface, which maintains appropriate tissue architecture while allowing necessary mobility.

The functional properties of GAGs within this fascial continuum extend beyond simple structural support. These molecules retain substantial water content, creating a hydrated gel-like environment that facilitates cell signaling and mechanotransduction processes essential for tissue homeostasis. The dynamic nature of this interface allows it to respond to inflammatory stimuli and coordinate tissue repair mechanisms through modulation of cytokine activity and cellular recruitment [93].

Pathological processes exploit the continuous nature of these fascial planes, following predictable anatomical patterns of spread. Endometriosis, malignant disease, and infectious processes disseminate along the GAG-rich fascial continuum via the paracolic gutters and peritoneal surfaces, respecting the architecture of these tissue planes [93,94,95]. Deep infiltrating endometriosis can completely obliterate the Pouch of Douglas, replacing the normal HA-GAG interface with fibrotic tissue that eliminates the physiological gliding planes between rectum and uterus [96,97,98].

Surgical approaches that recognize and preserve these fascial interfaces demonstrate superior functional outcomes. Nerve-sparing radical prostatectomy techniques that respect fascial gliding planes significantly reduce postoperative erectile and urinary dysfunction [99]. Similarly, nerve-sparing radical hysterectomy and total mesorectal excision procedures that maintain the integrity of fascial boundaries preserve autonomic innervation and reduce complications [93,100,101]. Understanding the Pouch of Douglas as a continuous GAG-rich fascial interface rather than an empty space fundamentally changes surgical strategy and improves patient outcomes.

#### 3.7.2. Ther Peritoneal Cavity

The peritoneal cavity further exemplifies the misconception of anatomical “emptiness.” Rather than a single void, the peritoneal cavity is a complex, fluid-filled space containing interconnected compartments formed by ligaments, mesenteries, and omenta, which create continuous pathways that direct fluid movement and disease spread. These structures divide the cavity into distinct anatomical and functional regions, such as the supramesocolic and inframesocolic compartments, with communication channels like the paracolic gutters facilitating fluid and cell transport [71,102]. The peritoneum itself is a dynamic serous membrane with epithelial and mesenchymal features, playing key roles in immune defense, tissue repair, and regulation of inflammatory responses through resident immune cells including T cells, B cells, NK cells, macrophages, and specialized milky spots in the omentum [70,103,104,105].

Imaging studies show that the peritoneal cavity is a potential space that becomes visible primarily when pathological processes, such as ascites or tumor metastasis, disrupt normal tissue relationships, creating separations detectable on scans [106]. See Table 2. The peritoneal vasculature and microenvironment adapt during disease states, with neoangiogenesis contributing to pathological progression, especially in peritoneal carcinomatosis [107]. Understanding the detailed anatomy and physiology of the peritoneal cavity is essential for accurate diagnosis, management of peritoneal diseases, and development of targeted therapies [72].

### 3.8. Universal HA-Mediated Gliding

These regional examples reveal a universal principle: specialized connective tissue organization enabling differential movement exists wherever gliding is required. Whether between organ mesenteries and peritoneal membranes, within synovial joints, between myofascial compartments, along neurovascular bundles, at tendon sheaths, beneath the skin in superficial fascia, or surrounding the spinal cord in the epidural space, these are all virtual spaces maintained through ongoing movement in continuous feedback loops. The thoracolumbar region exemplifies how fascial planes function as both structural boundaries and mobile interfaces.

The thoracolumbar fascia (TLF) is a complex connective tissue structure composed of multiple fascial planes that envelop and separate muscles in the lower back region. It includes distinct fascial planes such as the interfascial spaces between muscle groups. For example, the plane between the multifidus and longissimus muscles is targeted in thoracolumbar interfascial plane (TLIP) blocks used for pain control after spinal surgery [113,114]. Anatomically, the TLF consists of superficial and deep laminae forming retinacula over paraspinal muscles, with fascial sheaths like the paraspinal retinacular sheath and the lumbar interfascial triangle contributing to load transfer and muscle force distribution [112,115].

**Figure 4:**
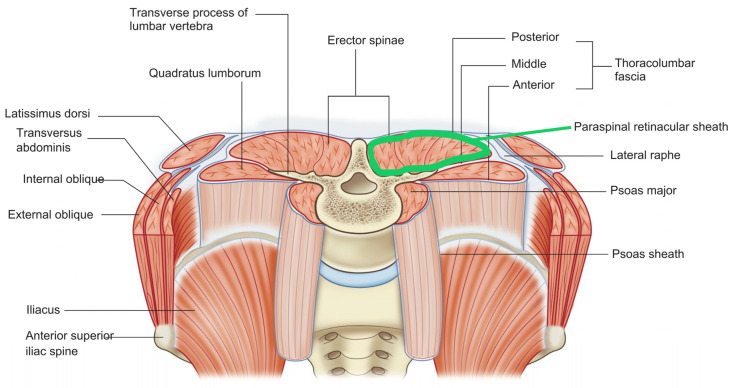
Thoracolumbar fascia (TLF) with the paraspinal retinacular sheath highlighted in green.

These fascial planes provide pathways for nerves and vessels and serve as targets for regional anesthesia techniques, highlighting their distinct anatomical and functional roles [116]. Ultrasound imaging studies confirm the presence of gliding mobility, which is important for normal tissue function and may be altered in pathological conditions [117]. The thoracolumbar fascia contains multiple fascial planes that are structurally and functionally significant in musculoskeletal biomechanics and clinical interventions. The paraspinal retinacular sheath (PRS) exemplifies the evolving understanding of fascial function. See Figure 4.

This deep sheath of the thoracolumbar fascia envelops the paraspinal muscles and acts as a hydraulic amplifier, supporting the lumbosacral spine by transmitting and distributing muscular forces [118]. Its biomechanical role in load transfer and lumbopelvic stability is well established, making it essential for maintaining spinal integrity during movement and static posture. Recent research interest has shifted from purely biomechanical models to biochemical mechanisms. Emerging studies highlight the potential importance of HA upregulation, calcium signaling, and the effects of reduced mobility on fascial pathology and low back pain, though direct clinical studies on these aspects in the PRS remain limited. This shift from biomechanical structure to biochemical dynamics represents the relationship: manifold fascial planes are HA-rich interfaces of heightened biochemical activity.

### 3.9. Calcium-HA Signaling: Molecular Mechanisms

The final aspect of our results shifts into the substrate of this biochemical environment: HA. Direct empirical evidence demonstrates that fascial planes throughout the body represent a unified system of HA-rich interfaces with varying gliding levels [1]. HA is highly responsive to chemical, mechanical, and hormonal changes, making it key to tissue adaptability [116]. HA binds specific cellular receptors, influencing cell survival, proliferation, adhesion, and migration. Its viscoelastic properties enable smooth internal movement by supporting mobility. Quantitative measurements confirm that HA concentration varies systematically with the degree of gliding movement required (Table 3). The manifold framework captures how this works spatially.

The fascial plane is a continuous biochemical surface—a manifold. HA concentration and mechanical properties vary smoothly across this space. Local cellular responses (opportunistic at the microscale) create gradients of HA and hydration across this manifold. These produce deterministic patterns at the macroscale. High-mechanical-stress regions develop higher HA concentrations, while low-stress regions maintain baseline levels.

These observations demonstrate that HA concentration correlates with functional gliding demands, raising the question: what are the specific cellular dynamics involved?

This question cannot be answered without introducing calcium ions (Ca^2+^), the body’s ubiquitous second messenger. Ca^2+^ influx triggers HA production, and the newly produced HA then modulates cellular responses. HA promotes cell migration and movement, reorganizes the cell’s internal skeleton (cytoskeleton), enables tissue gliding and flexibility, and triggers additional calcium signaling, perpetuating the cycle. This downstream effect of HA synthesis feeds back to influence the very calcium signaling that initiated HA production in the first place.

### 3.10. The Calcium-HAS2 (CHA) Feedback Loop: Molecular Mechanisms of Fascial Adaptation

#### 3.10.1. Mechanotransduction and HA Production: The CHA Cycle

**Figure 5:**
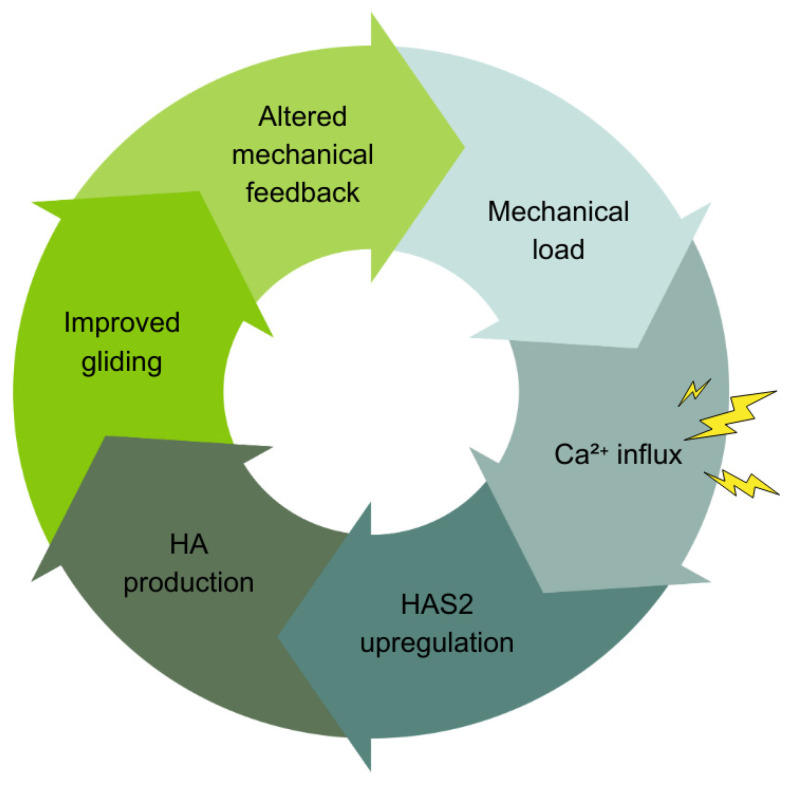
The Ca^2+^-HAS2 Axis (CHA). Mechanical loading triggers calcium ion (Ca^2+^) influx into cells through mechanosensitive channels. This activates intracellular signaling pathways such as CaMKII and ERK that regulate gene expression in mesenchymal cells like fasciacytes. This signaling cascade upregulates HAS2, the gene encoding hyaluronan synthase 2, which synthesizes HA. Mechanotransduction and ATP synthesis regulate HAS2 activity, linking mechanical or stress signals to HA synthesis. The result is a feedback loop where mechanical forces influence Ca^2+^ signaling, HAS2 expression, and HA synthesis, which in turn modulate tissue mechanics and cellular responses.

Based on well-established evidence, Kirkness and Scarlata (2025) recently identified this loop as the Ca^2+^-HAS2 Axis (CHA) [119]. See Figure 5. The framework describes how fascial tissues adapt rapidly to mechanical demands through autonomous, cell-intrinsic regulation. The CHA operates as follows: Mechanical load (movement and traction) deforms fasciacytes. This deformation opens mechanosensitive calcium channels—Piezo1 and TRPV4—embedded in cell membranes [120,121]. Calcium ions rush into the cell. These Ca^2+^ ions act as molecular switches, activating kinases like CaMKII and PKC. These kinases activate transcription factors (CREB and MAPK components) that turn on genes. Within hours, the HAS2 gene ramps up production of hyaluronan (HA). This newly produced HA absorbs an abundance of water. The significantly increased hydration supports the gel-like environment, reducing mechanical strain on cells. The reduced strain modulates calcium signaling, creating a self-regulating feedback loop.

The framework, extrapolating from evidence in fibroblasts and keratinocytes [122], posits that Ca^2+^ serves as the universal translator in fascia. It converts diverse stimuli—mechanical loading, tissue injury (ATP release), inflammation (cytokine signaling)—into coordinated HA production responses [123,124]. Individual Piezo1 or TRPV4 (ion) channels respond variably to mechanical stress [125], triggering Ca^2+^ influx that varies like a specific code. This ionic “message” is decoded by Ca^2+^-dependent kinases and transcription factors that regulate genes like HAS2. This drives HA synthesis within hours, consistent with the temporal precision of the CHA framework. This conceptualization aligns with established principles of Ca^2+^ signaling [122,126].

Negative feedback mechanisms maintain homeostasis and prevent overactivation. ATP breakdown to adenosine modulates the signaling. This Ca^2+^-mediated integration frames the stochasticity of fascia proposed in the present article. Individual cells respond variably based on local context, yet collective behavior produces coordinated field-level responses. Here, we apply CHA to understand how fascial planes thus function as manifolds of coordinated, body-wide activity via the HA substrate of fascial tissue.

#### 3.10.2. Mechanical Load-Calcium Signaling Regulates EMT/MET Plasticity and Cellular Phenotype

While the CHA regulates HA synthesis on a timescale of hours, calcium signaling exerts broader control over cellular identity itself. Specifically, HAS2 gene expression is increased, boosting HA synthesis [127,128]. The same mechanosensitive Ca^2+^ channels that drive HAS2 expression also regulate EMT/MET plasticity. Mechanical deformation activates these channels, leading to Ca^2+^ influx. This triggers downstream activation of Ca^2+^-dependent kinases and transcription factors. This cascade upregulates EMT markers such as N-cadherin and vimentin while downregulating epithelial markers like E-cadherin. E-cadherin acts like mortar binding bricks in a wall, while N-cadherin and vimentin free cells to move like mobile scouts.

Critically, this loop is bidirectional. Mechanical cues and matrix stiffness not only induce EMT via Ca^2+^ signaling but also modulate future mechanosensitivity and signaling through changes in ECM composition, such as increased HA synthesis and supporting dynamic EMT/MET transitions [39,129,130,131]. This Ca^2+^-dependent mechanotransduction pathway directly influences the cellular “Go or Grow” decisions, as it integrates mechanical and biochemical signals. Sustained or context-specific Ca^2+^ signaling can induce partial EMT in various cell types including cancer and epithelial cells [129,132,133,134,135,136,137]. This enables cells to exist in hybrid states with both migratory and proliferative potential—a plasticity crucial for cancer progression, tissue repair, and fascial adaptation [39,138].

This mechanochemical integration supports the CHA as a key regulator of fasciacyte phenotype, linking mechanical demands to cellular identity and function in health and disease. The resulting manifold—a continuously varying field of HA concentration, hydration, and mechanical compliance—arises from local cellular responses to their immediate environment. This organization is thus emergent. The empirical observations presented in our narrative synthesis provide the foundation for interpreting how these diverse phenomena integrate into a unified mechanistic framework.

## 4. Discussion

### 4.1. Interpreting the Evidence: Fascia as Stochastic Morphogenetic Field

We propose that the empirical observations presented in this narrative review support a new paradigm: fascia functions as a stochastic morphogenetic field. The quantitative correlation between HA concentration and gliding requirements (Table 3) demonstrates that fascial tissue organization adapts to functional demands. Imaging studies confirm that anatomical “spaces” represent potential interfaces rather than pre-existing voids (Table 2). Disease spread patterns along continuous fascial planes reveal underlying tissue connectivity. These observations suggest fascia operates as an integrated field of feedback rather than discrete anatomical structures.

Our perspective highlights the dual significance of EMT/MET plasticity. As we have reviewed, EMT plays crucial roles in embryonic development, tissue repair, regeneration, and cancer progression, with its deregulation contributing to tumor invasiveness and metastasis [40,41,50]. This transition is regulated by complex signaling pathways and transcription factors (e.g., Snail, Zeb1, Twist1) that orchestrate changes in gene expression, cytoskeletal remodeling, and cell adhesion, and also interact with epigenetic regulators to modulate cellular plasticity [139,140].

EMT is not a binary switch but involves intermediate hybrid states expressing both epithelial and mesenchymal markers, reflecting functional heterogeneity and plasticity within mesenchymal cell populations [141]. The reverse process, MET, is important for tissue regeneration and metastatic colonization, highlighting the reversible and context-dependent nature of these transitions [43,44]. EMT also influences the cancer immune microenvironment by promoting immunosuppression, which supports tumor progression and presents challenges and opportunities for targeted therapies combining EMT inhibition and immunotherapy [142,143].

In regenerative medicine, mesenchymal stromal/stem cells (MSCs) orchestrate tissue repair through secretory profiles, paracrine signaling, and ECM interactions. All of these are shaped by morphological state and field dynamics. These field effects mirror the CHA feedback loop, where local cellular responses integrate into global fascial organization. The clinical success of MSC-based therapies depends precisely on this principle: harnessing cells’ ability to sense, adapt, and respond to tissue environments through dynamic field interactions.

This convergence between our theoretical framework and clinical practice highlights the relevance of stochasticity for movement and manual therapists. Fascial health depends on supporting the dynamic, interconnected behavior of mesenchymal cells within their biochemical microenvironment. Regenerative therapies increasingly leverage this principle, harnessing mesenchymal plasticity by respecting tissue field dynamics rather than treating fascia as inert scaffolding [144,145]. The CHA framework provides molecular specificity to this clinical intuition. It identifies calcium-mediated HA regulation as a key mechanism through which fascial fields maintain homeostasis and respond to therapeutic intervention.

### 4.2. The Stochastic-Deterministic Complementarity: Manifolds as Emergent Structures

We propose that this calcium-mediated mechanotransduction reflects a fundamental principle. What appears as randomness at the microscale gives rise to predictable, organized patterns such as the fascial planes at the tissue scale. The evidence strongly supports that these fascial planes are continuous manifolds that enable the body-wide gel matrix to communicate.

The manifold framework may succeed precisely because it captures this emergent low-dimensional structure arising from high-dimensional stochastic processes (Table 4). Each fascial plane may represent a stable solution to the stochastic optimization problem posed by the body’s mechanical demands. This is an attractor state where probabilistic cellular exploration sculpts deterministic geometry. This apparent duality represents complementarity rather than contradiction. Stochasticity and determinism are two perspectives on the same biological reality.

We hypothesize that gliding maintains the system’s dynamic equilibrium through the calcium-HAS2 axis. Movement is not merely something that happens within the fascial manifold. It is what keeps the manifold viable, preventing degeneration into adhesion and dysfunction. The feedback loop operates through probabilistic cellular exploration that sculpts stable tissue-level architecture. This molecular evidence, drawn from multiple tissue types, suggests fascia functions as a calcium-regulated, self-organizing system.

### 4.3. Stochasticity Produces Macroscale Order That Constrains Microscale Stochasticity

At the cellular level, processes such as calcium channel opening and HAS2 gene expression occur probabilistically, leading to heterogeneous responses among fasciacytes in terms of HA production and mechanotransduction [148,149]. This stochasticity is influenced by local microenvironmental factors like matrix stiffness, existing HA content, and nutrient availability, which modulate cellular activity within the fascial plane. Fascia’s architecture is based on tensegrity principles, integrating mechanical stability with mobility through a continuous connective tissue network that self-organizes via genetic and epigenetic cues over time [34]. The presence of mechanoreceptors and fascial smooth muscle cells further supports fascia’s role as a responsive, self-regulating system connected to the nervous system, enabling dynamic changes in tissue viscosity and function.

Yet this apparently random cellular variability self-organizes into coordinated tissue-level function: enhanced tissue gliding. When integrated across cell populations, these opportunistic individual responses produce predictable tissue-level outcomes. HA concentration correlates systematically with functional gliding demands (Table 3). The mechanism operates through hydration lubrication. HA’s water-binding capacity creates viscoelastic properties that enable smooth tissue movement [61,150,151,152,153,154]. This represents emergence—deterministic patterns arising from stochastic processes.

### 4.4. Beyond “Friction” and “Layers”: Fascia as Dynamic Biochemical Landscape

We argue what is commonly described as “reducing friction” is biophysically more complex. HA’s hydration-dependent properties create boundaries, hydrated molecular films that separate and cushion tissue surfaces, preventing direct physical contact [150,152,155]. These hydrated films are not sliding surfaces; rather, their interface is an ion-mediated manifold relationship, reinforced by gliding. Interfaces enable viscoelastic buffering, where HA absorbs and dissipates mechanical energy, reducing resistance during movement [61]. The term “friction” serves as convenient shorthand for this complex ensemble. It encompasses hydration-dependent gliding, viscoelastic damping, and boundary layer effects—molecular and mechanical phenomena that we experience as smooth, low-resistance tissue gliding.

The manifold concept allows us to move beyond the implications of mechanistic “friction” between “layers” language. Instead, we understand fascia as a continuous, dynamically tensioned, self-regulating biochemical field. Rather than discrete layers that rub against each other, we see smoothly varying gradients. These include gradients of hydration, viscoelasticity, and mechanical compliance. Cells continuously adjust these properties through the CHA feedback loop in response to local mechanical demands. The fascial manifold is not a static structure. It is a living, self-organizing biochemical field where cells respond to—and reshape—their local environment.

### 4.5. CHA: Critical Knowledge Gaps and Clinical Relevance

#### 4.5.1. Unanswered Questions About the CHA Mechanism

Several key questions about CHA remain unanswered. Which mechanosensitive Ca^2+^ channels are expressed in fasciacytes, and at what levels? Are Piezo1 and TRPV4 the primary channels, or do other mechanosensors contribute? What is the precise time course between mechanical load and HAS2 upregulation in fascial tissue? What is the dose–response relationship? How does feedback regulation occur? Do elevated HA levels or reduced mechanical strain suppress Ca^2+^ signaling? If so, through what molecular mechanisms? Do different fascial regions (high vs. low gliding) show different CHA responsiveness? Does this correlate with baseline channel expression or signaling pathway sensitivity?

#### 4.5.2. Extrapolating Evidence from Related Cell Types

The authors have pointed out that direct evidence linking calcium signaling to HAS2 expression in fascial cells (fasciacytes) is currently lacking. We argue that this gap may reflect the recent recognition of fasciacytes as a distinct cell type rather than an absence of the mechanism itself. Research in other cell types—particularly keratinocytes and fibroblasts, which share mechanosensitive and HA-producing properties with fascial cells—has mapped the intermediary steps between calcium influx and HAS2 upregulation. This map shows a conserved signaling architecture that likely extends to fascia.

#### 4.5.3. Proposed Experimental Approaches to Test CHA

Direct testing in fascial tissues would address these gaps. We propose the following experiments:

**Calcium imaging under mechanical stimulation**: Controlled cyclic strain applied to fascial tissue is expected to induce rapid mechanotransduction responses, including a rise in intracellular Ca^2+^ within minutes, which can be detected using live-cell calcium imaging with indicators like Fluo-4. Although direct evidence in fascia is limited, mechanosensitive pathways such as Yes-associated protein (YAP) activation have been demonstrated in fascial fibroblasts following mechanical stimulation, supporting the plausibility of Ca^2+^-mediated signaling in these tissues [79]. Subsequent upregulation of HAS2 mRNA within a few hours and increased HA secretion over 6–8 h are consistent with the role of fasciacytes.

**Pharmacological blockade:** of mechanosensitive channels like Piezo1 or TRPV4, or chelation of intracellular calcium, would likely inhibit this mechanotransduction cascade, although such experiments have not yet been reported in fascia specifically. Regional differences in HAS2 expression and mechanosensitive channel abundance may exist, reflecting the functional diversity of fascial regions, but direct comparative data are currently lacking [156].

**In vivo movement studies**: Human subjects performing regular exercise versus sedentary controls should show differential HAS2 expression in accessible fascial planes (e.g., fascia lata biopsies). This should correlate with HA concentration measured biochemically and tissue mobility assessed by ultrasound elastography or manual palpation.

Overall, these proposed experiments align well with known mechanobiology principles and would fill important gaps in understanding how mechanical forces regulate HAS2 and HA production in fascial tissue.

#### 4.5.4. Clinical Relevance: HA Variations in Health and Disease

HA concentrations vary significantly across different tissues and physiological or pathological conditions. This influences diverse clinical phenomena. Elevated serum HA levels are associated with increased severity and poorer prognosis in diseases such as COVID-19. In COVID-19, higher HA correlates with inflammation, cytokine storm, and mortality risk [157,158]. In rheumatic diseases like rheumatoid arthritis, systemic sclerosis, and systemic lupus erythematosus, HA concentrations are increased.

These elevated levels correlate with disease activity and inflammation markers [158]. HA levels decline in osteoarthritis, affecting joint lubrication and viscoelasticity, which has led to its therapeutic use in managing this condition [159,160]. Additionally, HA concentrations in synovial fluid and tissues are linked to metabolic health, cardiovascular risk, and tissue repair processes. These variations are influenced by diet, gut microbiota, and systemic inflammation [161,162,163]. These regional and disease-specific alterations in HA underscore its role as both a biomarker and a therapeutic target in health and disease.

#### 4.5.5. CHA as a Stochastic-Deterministic Duality

These clinical observations are consistent with the CHA framework, though establishing causality requires the experimental approaches outlined above. The framework generates testable predictions that can be evaluated through controlled studies. Until such studies are completed, the CHA hypothesis remains a plausible but unproven explanation for fascial adaptation and dysfunction. In the meantime, the CHA illustrates the authors’ perspective: fascia functions as a stochastic-deterministic morphogenetic field.

### 4.6. MANIFEST: A Context-Flexible Nomenclature Tool

#### 4.6.1. Novel Contribution: Nomenclature for Biochemical Dynamics

This paper’s novel contribution lies in recognizing that advancing fascial research—particularly understanding its biochemical environment—requires appreciating fascia’s stochastic-deterministic dual nature. This recognition creates a nomenclature challenge: traditional anatomical language was developed for static, stratified structures. Yet research demonstrates that fascia operates as a continuous manifold where ‘layers’ do not truly exist as discrete entities. What we are taught as layers are in fact regions of differentiated organization within a unified field showing variable HA concentrations and calcium-responsive cell populations.

However, dismissing terms like ‘layers’ or ‘superficial versus deep fascia’ would be pedagogically counterproductive. These terms remain useful in specific contexts: teaching beginners, providing surgical landmarks, or communicating with clinicians trained in traditional frameworks. The challenge is not choosing between old and new terminology, but providing a framework that enables practitioners to use appropriate language for each context while understanding the underlying biochemical reality. MANIFEST (Table 5) addresses this by offering context-flexible nomenclature that bridges traditional anatomical description with emerging biochemical understanding.

#### 4.6.2. From Structural Layers to Biochemical Manifolds: Why New Language Is Necessary

The growing body of research on fascial biochemistry—HA dynamics, calcium signaling, mechanotransduction, stochastic cellular organization—reveals properties that traditional nomenclature cannot capture. Table 3 demonstrates that HA concentration varies systematically with gliding requirements. Our proposed calcium-HAS2 axis explains how self-organizing tissue interfaces emerge from this biochemical regulation—a morphogenetic field, fundamentally different from classical anatomical ‘layers.’

The stochastic-deterministic duality central to our framework means fascia cannot be adequately described by purely structural terms (‘the thoracolumbar fascia consists of anterior, middle, and posterior layers’) or purely functional terms (‘fascia transmits force’). We need language capturing how probabilistic molecular events (stochastic Ca^2+^ signaling, variable HAS2 expression, heterogeneous HA secretion) generate predictable tissue-level organization (deterministic fascial planes with characteristic HA concentrations).

Manifold topology provides this language. ‘Fascial manifolds’ captures the mathematical reality: locally differentiated but globally continuous. ‘Persistent morphogenetic field’ captures the developmental reality: embryonic organization continuing throughout life. ‘Fascial planes’ captures the clinical reality: reliable anatomical interfaces. MANIFEST enables discussion of calcium-regulated biochemical dynamics while maintaining practical anatomical terminology—each serving appropriate contexts.

#### 4.6.3. MANIFEST in Practice: Navigating Between Frameworks

MANIFEST does not replace standardized anatomical definitions such as those proposed by Stecco et al. (2025) [1]. Standardization serves essential purposes: research reproducibility, clinical communication, educational consistency. Rather, MANIFEST functions as a flexible framework enabling the field to advance while maintaining standardization where appropriate. It is an appropriately interpretative tool arising in recognition of a fundamentally interpretative tissue. Three vignettes illustrate how MANIFEST enables the field to advance while maintaining pedagogical accessibility and clinical utility:

*Anatomy education:* An instructor teaches first-year students: ‘Separate the superficial fascia from the deep fascia’—essential terminology for learning dissection technique. In a graduate seminar, the same instructor explains: ‘Stratified layers do not exist in fascial manifolds. What we call superficial versus deep fascia are regions of a continuous tissue field showing different HA content and cellular organization. The “separation” you perform is an artificial dissection of a natural continuum—useful pedagogically, but not biologically fundamental.’ This progression from operational terminology (*fasciae*, *fascial planes*) to underlying reality (*fascial manifolds*, *morphogenetic fields*) exemplifies MANIFEST’s flexibility. Neither level is ‘wrong’—they serve different educational purposes.

*Research communication:* A researcher studying the biochemical environment of the thoracolumbar fascia finds traditional ‘posterior layer’ and ‘anterior layer’ terminology inadequate for describing continuous HA gradients and calcium-responsive cell populations distributed across the entire region. Using MANIFEST: anatomy journals receive descriptions using *fascial planes* (maintains literature continuity); biochemistry conferences hear about *fascial matrix* (emphasizes molecular mechanisms); clinical presentations discuss *fascial network* (captures integrated function); theoretical papers use *fascial manifold* framework (enables mathematical modeling of HA dynamics). Each context receives appropriate language while the underlying biochemical data—and its stochastic-deterministic nature—remain consistent.

*Clinical translation:* Understanding fascial dysfunction through the CHA framework requires new language. Traditional description: ‘adhesions between fascial layers.’ Biochemical understanding: ‘reduced gliding in fascial interfaces due to decreased HA production from inadequate mechanical stimulation of calcium-HAS2 signaling.’ The first implies structural damage; the second suggests biochemical dysfunction potentially reversible through movement therapy. MANIFEST provides terminology (*fascial interfaces*, *morphogenetic field dynamics*) enabling clinicians to communicate biochemical mechanisms alongside traditional structural concepts.

### 4.7. Future Research Opportunities

Clinical practice has long recognized the importance of fascial continuity in tissue organization. A new paradigm is emerging that views fascia as morphogenetic, actively guiding tissue development, organization, and repair throughout life. The gel-like HA continuum is an ionotropic medium, fertile ground for exploring links to consciousness. This represents an evolution from understanding fascia as “merely” continuous to recognizing it as an adaptive field orchestrating complex biological processes. Future research should elucidate specific molecular mechanisms linking calcium signaling to HAS2 regulation in mesenchyme, investigate how chronic pathway disruption contributes to pathology, and develop targeted therapeutic interventions restoring healthy HA homeostasis.

## 5. Conclusions

This perspective highlights four conclusions: (1) anatomical “virtual spaces” are HA-tissue manifolds tightly coupled with calcium coordination; (2) fascia functions as a stochastic morphogenetic field where deterministic patterns emerge that are clinically and educationally relevant; (3) the MANIFEST tool provides context-flexible fascial nomenclature; (4) hermeneutic approaches enable synthesis across theoretical domains.

This synthesis integrates manifold topology, stochastic organization theory, calcium signaling and morphogenetic field concepts to reconceptualize fascial anatomy as a dynamic, self-organizing continuum. This perspective unifies these diverse frameworks into a cohesive model that challenges traditional anatomical nomenclature while maintaining clinical relevance. The emerging evidence surrounding the calcium–HAS2 relationship exemplifies fascia’s dual nature. Its stochastic cellular variability gives rise to deterministic structural organization. This underscores the interplay between microscopic dynamics and macroscopic form.

The MANIFEST framework complements this narrative synthesis. It provides a context-flexible nomenclature tool that supports conceptual coherence across research, clinical, and educational domains. Our hermeneutic approach aligns with the standards of qualitative rigor established in the medical education literature. This Perspective advances a cross-disciplinary model of fascia that connects molecular processes, embodied anatomy, and reflective pedagogy within a narrative synthesis.

## Figures and Tables

**Table 1:** EMT/MET stochasticity and morphogenetic field activity in fascia and mesenchyme. This framework establishes that fascial tissues inherently operate through stochastic cellular processes—variable EMT/MET transitions, probabilistic cell fate decisions, and fluctuating microenvironmental signals—that self-organize into deterministic tissue-level patterns.

Process/Field Activity	Role in Fascia/Connective Tissue	Stochastic Features and Manifold Principle
EMT/MET	Drives cell migration, differentiation, tissue remodeling, and repair; persists beyond embryogenesis in fascia and loose areolar tissue as morphogenetic field activity [49,50]	Variable gene expression, intermediate/hybrid cell states, probabilistic cell fate decisions, and context-dependent transitions; non-binary, plastic, and influenced by microenvironmental fluctuations [39,49,51,52]
ECM Interaction and Mechanotransduction	Guides cell fate, organization, and collective migration; mechanical cues (e.g., mesoderm stiffening) can trigger EMT in overlying cells, exemplifying stochastic morphogenetic responses [49,50,52]	Microenvironmental signal variability, dynamic feedback, and emergent deterministic patterns from probabilistic cellular responses [39,49,50,52]
Morphogenetic Field Dynamics	Mesenchyme and loose areolar tissue act as dynamic morphogenetic fields, integrating stochastic fluctuations into coordinated tissue patterning [49,50]	Stochastic mechanical and signaling fluctuations drive coordinated morphogenesis and collective migration (e.g., neural crest migration) [49,50]

**Table 2:** Imaging modalities demonstrate the peritoneal cavity as a potential space that becomes visible only under pathological conditions disrupting normal tissue relationships.

Imaging Modality	Normal Appearance	Pathological Appearance (Space Visible)	Citations
CT/MRI	Not visible, layers apposed	Fluid, gas, or masses separate layers	[108,109]
Ultrasound	Not visualized	Ascites or collections seen	[109,110]
Nuclear Medicine	Not visualized	Abnormal tracer in fluid collections	[109,111,112]

**Table 3:** Gliding and hyaluronan from Fede et al. (2018) [66].

Anatomical Site	HA Concentration (μg/g)	Gliding Requirement
Ankle retinacula (joint fascia)	90	High
Fascia lata	35	Moderate
Rectus sheath	29	Moderate
Fascia over trapezius/deltoid	6	Low

**Table 4:** Stochasticity vs. Determinism in Fascia and Connective Tissue [146,147].

Aspect/Model	Stochastic Features	Deterministic Features
Molecular Turnover (e.g., HA, FA proteins	Captures random entry/exit, turnover, and noise in molecular interactions; explains cell-to-cell variability and dynamic adaptation	Uses average rates and fixed parameters to predict mean behavior over time
Tissue Damage/Remodeling	Accounts for variability in fiber length, rupture, and repair; models heterogeneity in response to stress	Predicts overall softening, damage, and adaptation under given loading conditions
Population/Cellular Response	Explains adaptation to stress via random cell-to-cell differences; important at low stress or in small populations	Deterministic effects dominate at higher stress or in large populations; can reveal system-level stability and control
Functional Connectivity	Dynamic fluctuations and random walks in tissue connectivity, especially with aging	Underlying network structure and mean connectivity remain ordered and stable
Tissue Morphogenesis	Random reaction-diffusion and noise-driven morphogenesis lead to emergent, complex tissue patterns	Deterministic systems yield predictable, stable tissue structures under typical conditions
Predictive Power	Reveals hidden structures, noise, and rare events; essential for understanding heterogeneity and resilience	Provides clear, reproducible predictions and system-level insights; useful for control and intervention

**Table 5:** MANIFEST Context-Specific Adaptations to Fascial Terminology and Nomenclature.

Term	Context	Rationale	Appropriate Use	Acronym
Persistent Morphogenetic Field	Theoretical/Research	Highest-level conceptual framework	When discussing fascia’s role in development, adaptation, and continuous tissue organization across the lifespan	Morphogenetic
Fascial Manifolds	Mathematical/Clinical Anatomy	Replaces “virtual spaces” with updated geometric terminology	When describing continuous tissue interfaces and anatomical “spaces”	Anatomical
Fascial Network	Clinical Practice	Emphasizes connectivity	When describing tissue relationships in manual therapy, surgery, or rehabilitation contexts	Network
Fascial System	Physiological Education	Systems-level pedagogy	When teaching integrated body systems and need familiar educational framework for students	Integrative (integrates all systems)
Fasciae	Laboratory/Dissection	Discrete structural identification	When anatomical dissection requires naming specific fascial structures (e.g., “the thoracolumbar fasciae”)	Finite (refers to discrete, countable fascial structures)
Fascial Continuum	Movement/Manual Therapy	Highlights unbroken tissue flow	When explaining force transmission, movement patterns, or myofascial treatment approaches	Education
Fascial Matrix	Structural/Biomechanical Research	Structural and mechanical properties	When discussing ECM, mechanical properties, or tissue engineering applications	Synergy
Fascial Planes	Therapeutic/Clinical/Surgical	Established terminology in clinical navigation	When describing surgical approaches, anesthetic blocks, or anatomical tissue interfaces	Therapeutic and Clinical

## Data Availability

No new data were created or analyzed in this study. Data sharing is not applicable to this article.

## Abbreviations

Ca^2+^	Calcium ions
CHA	Calcium-HAS2 axis (also Ca^2+^-HAS2 Axis)
ECM	Extracellular matrix
EMT	Epithelial–mesenchymal transition
HA	Hyaluronic acid (also hyaluronan)
HAS2	Hyaluronan synthase 2
MANIFEST	Morphogenetic, Anatomical, Network, Integrative, Finite, Education, Synergy, Therapeutic and Clinical (context-flexible nomenclature framework)
MET	Mesenchymal–epithelial transition
MSCs	Mesenchymal stem cells/Mesenchymal stromal/stem cells
